# Supplementary material for: Hybrid Models and Biological Model Reduction with PyDSTool
Source: PLoS Comput Biol. 2012 Aug 9;8(8):e1002628. doi: 10.1371/journal.pcbi.1002628 (PMC3415397; doi:10.1371/journal.pcbi.1002628)
Supplement: Text S4 — Complete source code for the PyDSTool package (version 0.88.120504). Includes API documentation and help files linking to web pages. This file is identical to the current public release on Sourceforge.net. (ZIP) [file pcbi.1002628.s004.zip › PyDSTool/html/PyDSTool.Toolbox.dssrt.join_fast-class.html]

xml version="1.0" encoding="ascii"?


PyDSTool.Toolbox.dssrt.join\_fast


| Home | Trees | Indices | Help | PyDSTool |
| --- | --- | --- | --- | --- |

Package PyDSTool ::
Package Toolbox ::
Module dssrt ::
Class join\_fast

[hide private]

[frames] | no frames]

# Class join\_fast

source code

```
      object --+    
               |    
common.predicate --+
                   |
                  join_fast
```

---


|  |  |
| --- | --- |
| Instance Methods | [hide private] |

|  |  |
| --- | --- |
| precondition(self, epochs)  Override if needed | source code |

|  |  |
| --- | --- |
| evaluate(self, epoch) | source code |

**Inherited from `common.predicate`**:
`__call__`,
`__init__`

**Inherited from `object`**:
`__delattr__`,
`__getattribute__`,
`__hash__`,
`__new__`,
`__reduce__`,
`__reduce_ex__`,
`__repr__`,
`__setattr__`,
`__str__`


|  |  |
| --- | --- |
| Class Variables | [hide private] |

name = `'join_fast'`


|  |  |
| --- | --- |
| Properties | [hide private] |

**Inherited from `object`**:
`__class__`


|  |  |
| --- | --- |
| Method Details | [hide private] |

|  |  |
| --- | --- |
| precondition(self, epochs) | source code |

Override if needed

Overrides: common.predicate.precondition: *(inherited documentation)*

|  |  |
| --- | --- |
| evaluate(self, epoch) | source code |

Overrides: common.predicate.evaluate

  


| Home | Trees | Indices | Help | PyDSTool |
| --- | --- | --- | --- | --- |

|  |  |
| --- | --- |
| Generated by Epydoc 3.0.1 on Fri May 4 15:24:08 2012 | http://epydoc.sourceforge.net |
